# Micronutrient status among women in the midlife in selected Asian regions: a scoping review

**DOI:** 10.3389/fgwh.2026.1729971

**Published:** 2026-05-21

**Authors:** Unnop Jaisamrarn, Premitha Damodaran, Maria Antonia Habana, Sajidah Banu, Arya Shah, Vandana Garg, Sheryl Tan

**Affiliations:** 1 Center of Excellence in Menopause and Aging Women Health, Faculty of Medicine, Chulalongkorn University, Bangkok, Thailand; 2 Pantai Hospital Kuala Lumpur, Kuala Lumpur, Malaysia; 3 College of Medicine, University of the Philippines Manila, Manila, Philippines; 4 Haleon, Singapore, Singapore; 5 Tech Observer Asia Pacific, Singapore, Singapore

**Keywords:** Asia, menopause, micronutrient status, midlife women, nutritional intake

## Abstract

**Introduction:**

Micronutrient status is critical for the health and well-being of midlife women during the menopausal transition. However, data on micronutrient intake and status among midlife Asian women remain limited. This review examined the landscape of micronutrient intake, deficiency, and health impact in this population.

**Methods:**

Using a scoping review approach guided by a Population-Concept-Contextframework, we descriptively synthesized evidence from population-level and peer-reviewed data across six Asian regions (India, Indonesia, Japan, the Philippines, South Korea, and Taiwan). The review focuses on B-vitamins, calcium, vitamin D, and magnesium, due to their roles in metabolic, musculoskeletal, cognitive, and mental health.

**Results:**

Eighty-six publications and ten reports were identified. Despite methodological differences, evidence consistently indicated suboptimal intakes or deficiency. Available data suggested associations between adequate intake or status and better bone health, metabolism, cognition, and quality of life.

**Discussion:**

Micronutrient inadequacy among this population is common yet under-recognized. Strengthening surveillance and developing targeted interventions are critical to support healthy aging and disease prevention among women in midlife and beyond.

## 1 Introduction

Micronutrients are essential for maintaining health and well-being, especially for women in midlife, which encompasses perimenopause, the menopausal transition, and early post-menopause. While the timing of the menopausal transition varies between individuals, this life stage generally corresponds to women approximately 40–65 years of age, during this life stage, progressive estrogen decline and biological aging (1, 2) are accompanied by hormonal fluctuations, reductions in basal metabolism and shifts in body composition and fat distribution (3). These changes profoundly affect women's health needs, health outcomes, and well-being. Adequate micronutrient intake and maintaining a healthy nutritional status have been associated with mitigating the impact of aging and pathophysiological processes, such as chronic inflammation, on metabolic, musculoskeletal, cognitive, and mental health (4–7).

Addressing nutritional gaps aligns with the United Nations' Sustainable Development Goal 2 (SDG-2) to end all forms of malnutrition by 2030. Ensuring adequate micronutrient intake is not only essential for individual health but also contributes to broader public health efforts to reduce malnutrition and promote well-being across the lifespan (8). Despite the recognized importance of micronutrients for healthy aging (9–11), midlife women remain an underrepresented demographic in this field of research (12). This gap is especially concerning in Asia, a region now experiencing rapid population aging associated with the demographic transition (13). In Asia, women have a life expectancy of around five years longer than men, on average, but spend 25% more time in poor health (13, 14). It is unclear how prepared women in Asia are for the physical, psychological, and endocrine changes occurring during this life stage, specifically in terms of optimal nutrition. Thus, it is crucial to understand their current nutritional status and health needs, even more so given the paucity of regional data (15).

We therefore examined population-level data on micronutrient intake and status/deficiency across six Asian regions. We focused on micronutrients associated with chronic disease risk and/or health conditions relevant to midlife/menopause: B-group vitamins, calcium, vitamin D, and magnesium (3, 16–18). We also reviewed published evidence on associations between these micronutrients and key health domains relevant to midlife women, including metabolic health, musculoskeletal health and mobility, and cognitive/mental health.

Given the heterogeneity of available evidence across Asia, a scoping review approach was selected for this study. Data on micronutrient intake and status among midlife women vary widely in study design, population definitions, biomarkers assessed, and reporting formats, and include both peer-reviewed publications and population-level surveillance reports. A scoping review is therefore appropriate to map the breadth of existing evidence, identify key data gaps, and synthesise diverse sources that are not amenable to quantitative synthesis within a formal systematic review framework.

This comprehensive overview of micronutrient status among midlife women in Asia highlights areas where further research is urgently needed. Addressing these gaps may help inform nutritional and public health interventions to support the overall health and well-being of women throughout midlife and the menopausal transition.

## 2 Methods

### 2.1 Scoping review approach: target concepts, research questions, and targeted literature search strategy

This scoping review was developed to provide a descriptive synthesis of the current landscape of micronutrient intake, status, and health outcomes among midlife women in Asia. The review was conducted in accordance with Preferred Reporting Items for Systematic reviews and Meta-Analyses-Scoping Reviews (PRISMA-ScR) guidance. The research questions addressed in this review were:

1. What is known about micronutrient intake and micronutrient status (deficiency or insufficiency) among midlife women in Asia?
2. What is the health impact of micronutrient intake deficiency or insufficiency in midlife women in Asia?

The Population, Concept and Context (PCC) framework and the research questions were used to define the eligibility criteria for this review. The population of interest included midlife women (peri-menopausal or post-menopausal, corresponding to an age range of approximately 40–65 years) in 6 regions of Asia (India, Indonesia, Japan, the Philippines, South Korea, and Taiwan). The regions included in this review were selected *a priori* to provide representation across different Asian subregions and development contexts. Since it is estimated that perimenopause onset generally occurs around age 40–45 but can vary considerably, the population age range of interest potentially includes the 35–40 age group as well as those aged 40–55. These criteria were used to select publications and reports representing populations across the Asian region at various levels of socioeconomic development and stages of the demographic transition, supporting a comprehensive understanding of how differing socio-economic contexts may influence micronutrient intake and health outcomes in midlife women.

The micronutrients of interest were B-group vitamins, calcium, vitamin D, and magnesium. Health outcomes of interest included those important for women in mid-life. Details are provided in Supplementary Table S1.

### 2.2 Identification of national-level data on micronutrient recommendations, intake, and status (research question 1)

Internet searches were performed to identify relevant, publicly available reports containing national-level data, either published or hosted by national health authorities and government bodies, or non-governmental organizations in the Asian regions of interest. Data types of interest were: intake recommendations for micronutrients of interest, intake data, and micronutrient status or biomarker data (e.g., serum levels, prevalence of deficiency) for the population of interest. Reports that did not contain micronutrient intake/status data reported separately for women/men and by age group were excluded from consideration. As national survey and government report data were typically presented using age-banded categories rather than menopausal stage; these data were interpreted as proxies for midlife women, recognising that age bands do not always map precisely to menopausal status.

### 2.3 Identification of relevant peer-reviewed publications (research question 2)

We searched for peer-reviewed publications reporting primary research or systematic reviews of primary research relevant to the research question: evaluating (a) participants' intake or status for the micronutrients of interest; (b) relationships between micronutrient intake/status and health outcomes of interest. A structured search was performed in PubMed (date range: 01-Jan-2013 to 13-Aug-2024) to retrieve publications with data on the target topics for this review. PubMed was selected as the primary biomedical database due to its relevance to the research focus on health outcomes. The search strategy is detailed in Supplementary Table S2. An example search string is given for one micronutrient: “(Magnesium[Title/Abstract] OR Magnesium[Mesh] OR “Magnesium Deficiency”[Mesh]) AND (“Nutritional Status”[Mesh] OR “Diet”[Mesh] OR “Dietary Supplements”[Mesh] OR (dietary intake) OR status OR deficiency OR insufficiency OR (serum concentration) OR (plasma concentration) OR (serum level) OR (blood concentration)) AND (“Perimenopause” [MeSH Terms] OR “Menopause”[MeSH Terms] OR “Postmenopause”[MeSH Terms] OR “perimenopaus*”[All Fields] OR “menopaus*”[All Fields] OR “postmenopaus*”[All Fields]) AND “female”[MeSH Terms] AND (“Asia”[Mesh] OR China OR Japan OR Korea OR India OR Indonesia OR Malaysia OR Philippines OR Singapore OR Taiwan OR Thailand OR Vietnam OR “Southeast Asia” OR “East Asia” OR “South Asia” OR “Asia Pacific”) AND (“metabolic syndrome” OR “cardiovascular disease” OR obesity OR dyslipidemia OR diabetes OR osteoporosis OR osteoarthritis OR (cognitive function) OR cognition OR anxiety OR depression OR “sleep disorder” OR “quality of life” OR (menopausal symptoms))”.

### 2.4 Publication screening, selection, and data extraction

Three reviewers performed an initial screening to shortlist retrieved records based on titles/abstracts and screened shortlisted full-text publications to decide their inclusion/exclusion in the review. Disagreements were resolved by discussion among reviewers. Randomized controlled trials and observational studies (cohort or longitudinal, cross-sectional or surveys) reporting data for women in midlife were included. Eligible studies included those reporting on peri-menopausal and or post-menopausal populations, as well as studies defining midlife women using age-based cohorts. We focused on studies in generally healthy individuals, excluding studies in institutionalized individuals or with terminal, serious, or life-threatening conditions. Non-English publications, animal studies, and those outside the scope of interest were excluded (eligibility criteria are listed in Supplementary Table S1). Data on study objectives, location, main outcomes analyzed, and key findings relevant to the research questions were extracted from the included publications and presented descriptively (Supplementary Table S3**)**. Consistent with PRISMA scoping review methodology, no formal risk of bias assessment was undertaken.

### 2.5 Synthesis of findings

This review synthesized data from published studies and national-level reports, providing an overview of micronutrient intake and status (deficiency/insufficiency) in midlife women. We focused on current dietary recommendations, actual intake, and status of these micronutrients in the populations examined, and relationships between micronutrient intake/status and health outcomes.

## 3 Results

### 3.1 Peer-reviewed publications and population-level reports included in the review

**Figure 1:**
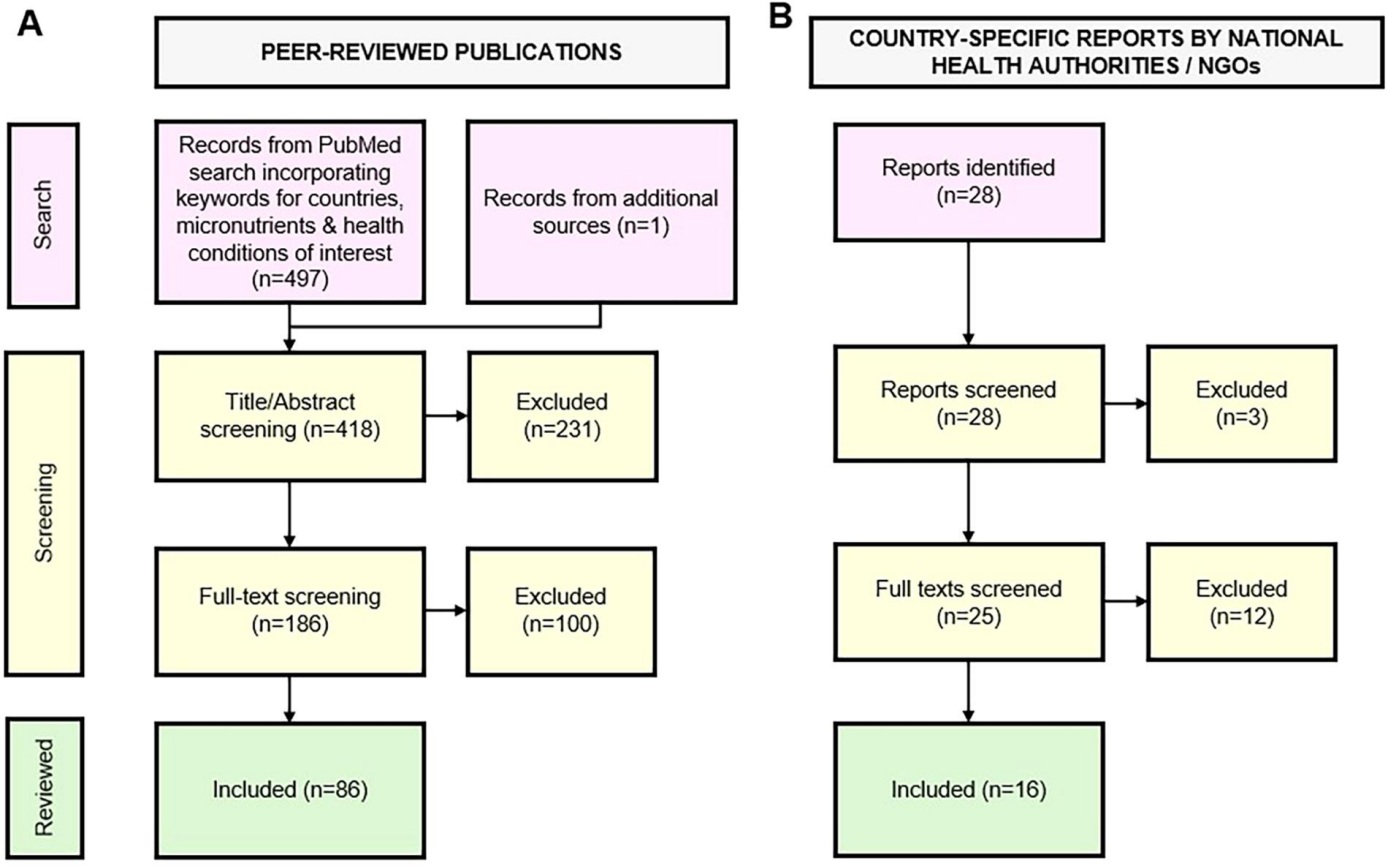
Study selection flow diagram. (**A**) Identification, screening, and inclusion of peer-reviewed publications retrieved from PubMed and additional sources. (**B**) Identification, screening, and inclusion of country-specific population-level reports from national health authorities and non-governmental organizations (NGOs).

Eighty-six peer-reviewed publications containing data relevant to the research questions were identified **(**Figure 1A). We identified a total of 10 reports containing population-level data on intake, status, or prevalence of deficiency for the micronutrients and population of interest (Figure 1B). The Results present a descriptive mapping of the available evidence on micronutrient intake and status among midlife women in Asia, including reported associations with health outcomes.

### 3.2 Micronutrient recommendations

Table 1 presents intake recommendations by region for each micronutrient of interest. These dietary reference intake (DRI) values include adequate intake (AI), recommended nutrient intake (RNI), recommended adequacy amount (Indonesia only), and recommended dietary allowance (RDA). Among the DRI values, RDAs and RNIs are considered more comparable; thus, comparisons are limited to these parameters.

**Table 1:** Dietary reference intake values for B-group vitamins, calcium, vitamin D, and magnesium, by region.

Region	Age group	Vitamin B1	Vitamin B2	Vitamin B3	Vitamin B6	Vitamin B9	Vitamin B12	Calcium	Vitamin D	Magnesium
Taiwan (19)	19–30	0.9 mg *	1.0 mg *	14 mg NE *^1^	1.5 mg *	400 μg *	2.4 μg †^a^	1,000 mg †^a^	10 μg †^a^	320 mg †
	31–50	0.9 mg *	1.0 mg *	14 mg NE *^1^	1.5 mg *	400 μg *	2.4 μg †^a^	1,000 mg †^a^	10 μg †^a^	320 mg †
	51–70	0.9 mg *	1.0 mg *	14 mg NE *^1^	1.6 mg *	400 μg *	2.4 μg †^a^	1,000 mg †^a^	15 μg †^a^	310 mg †
	71+	0.9 mg *	1.0 mg *	14 mg NE *^1^	1.6 mg *	400 μg *	2.4 μg †^a^	1,000 mg †^a^	15 μg †^a^	300 mg †
Philippines (20)	19–29	1.1 mg ‡	1.1 mg ‡	14 mg NE ‡^2^	1.3 mg ‡	400 μg DFE ‡^6^	2.4 μg ‡	750 mg ‡	5 μg †	210 mg ‡
	30–49	1.1 mg ‡	1.1 mg ‡	14 mg NE ‡^2^	1.3 mg ‡	400 μg DFE ‡^6^	2.4 μg ‡	750 mg ‡	5 μg †	210 mg ‡
	50–59	1.1 mg ‡	1.1 mg ‡	14 mg NE ‡^2^	1.6 mg ‡	400 μg DFE ‡^6^	2.4 μg ‡	800 mg ‡	10 μg †	210 mg ‡
	60–69	1.1 mg ‡	1.1 mg ‡	14 mg NE ‡^2^	1.6 mg ‡	400 μg DFE ‡^6^	2.4 μg ‡	800 mg ‡	15 μg †	210 mg ‡
	70+	1.1 mg ‡	1.1 mg ‡	14 mg NE ‡^2^	1.6 mg ‡	400 μg DFE ‡^6^	2.4 μg ‡	800 mg ‡	15 μg †	210 mg ‡
Indonesia § (21)	19–29	1.1 mg	1.1 mg	14 mg	1.3 mg	400 μg	4.0 μg	1,000 mg	15 μg	330 mg
	30–49	1.1 mg	1.1 mg	14 mg	1.3 mg	400 μg	4.0 μg	1,000 mg	15 μg	340 mg
	50–64	1.1 mg	1.1 mg	14 mg	1.5 mg	400 μg	4.0 μg	1,200 mg	15 μg	340 mg
	65–80	1.1 mg	1.1 mg	14 mg	1.5 mg	400 μg	4.0 μg	1,200 mg	20 μg	320 mg
	80+	1.1 mg	1.1 mg	14 mg	1.5 mg	400 μg	4.0 μg	1,200 mg	20 μg	320 mg
Japan (22)	18–29	1.1 mg ¶	1.2 mg ¶	11 mg NE ^4^	1.2 mg ¶	240 μg ¶	2.4 μg ¶	650 mg ¶	5.5 μg †	270 mg ¶
	30–49	1.1 mg ¶	1.2 mg ¶	12 mg NE ^4^	1.2 mg ¶	240 μg ¶	2.4 μg ¶	650 mg ¶	5.5 μg †	290 mg ¶
	60–69	1.0 mg ¶	1.1 mg ¶	11 mg NE ^4^	1.2 mg ¶	240 μg ¶	2.4 μg ¶	650 mg ¶	5.5 μg †	290 mg ¶
	70+	0.9 mg ¶	1.1 mg ¶	10 mg NE ^4^	1.2 mg ¶	240 μg ¶	2.4 μg ¶	650 mg ¶	5.5 μg †	270 mg ¶
S. Korea (23)	19–29	1.1 mg ‡	1.2 mg ‡	14 mg NE ‡^3^	1.4 mg ‡	400 μg DFE ‡^7^	2.4 μg ‡	700 mg ‡	10 μg †	280 mg ‡
	30–49	1.1 mg ‡	1.2 mg ‡	14 mg NE ‡^3^	1.4 mg ‡	400 μg DFE ‡^7^	2.4 μg ‡	700 mg ‡	10 μg †	280 mg‡
	50–64	1.1 mg ‡	1.2 mg ‡	14 mg NE ‡^3^	1.4 mg ‡	400 μg DFE ‡^7^	2.4 μg ‡	800 mg ‡	10 μg †	280 mg ‡
	65–74	1.0 mg ‡	1.1 mg ‡	13 mg NE ‡^3^	1.4 mg ‡	400 μg DFE ‡^7^	2.4 μg ‡	800 mg ‡	15 μg †	280 mg ‡
	75+	0.8 mg ‡	1.0 mg ‡	12 mg NE ‡^3^	1.4 mg ‡	400 μg DFE ‡^7^	2.4 μg ‡	800 mg ‡	15 μg †	280 mg ‡
India ¶ (24)	Adult women (18+)	1–1.4 mg ^b^	1.1–1.7 mg ^b^	12–16 mg NE ^b,2^	2.0 mg	200 μg	1.0 μg	600 mg	NA ^c^	310 mg
	Post menopausal women	–	–	–	–	–	–	800 mg	–	–

*Dietary Reference Intakes (DRIs).

† Adequate Intake (AI).

‡ Recommended Nutrient Intake (RNI).

§ Values for Indonesia represent recommended adequacy amounts.

¶ Recommended Dietary Allowance (RDA).

¶ Values for India are provided as Recommended Dietary Allowances (RDA).

a. Recommended values for both males and females, not differentiated by gender.

b. Values presented as ranges reflect varying requirements based on levels of physical activity: sedentary, moderate, and heavy.

c. Not available: The Indian Council of Medical Research (ICMR) does not provide an RDA value for vitamin D for the Indian population. The ICMR recommends that adequate outdoor exposure should serve as the primary source of vitamin D. In cases of minimal sun exposure, a daily intake of 10 µg of vitamin D is recommended.

1. NE: Niacin Equivalent is nicotinic acid equivalent. Nicotinoids include nicotinic acid and nicotinic acid amide, expressed in nicotinic acid equivalents.

2. NE: As Niacin Equivalent.

3. NE: Niacin Equivalent, where 1 mg NE = 1 mg niacin = 60 mg tryptophan.

4. NE: Niacin Equivalent = Niacin + 1/60 Tryptophan.

5. NE: Niacin Equivalent where, 1 niacin equivalent = 1 mg available nicotinic acid or 60 mg tryptophan.

6. DFE: 1 dietary folate equivalent = 1 µg food folate = 0.6 µg folic acid from fortified foods or as supplement = 0.5 µg taken on an empty stomach.

7. DFE: Dietary Folate Equivalents.

We noted variations in recommended intake within national dietary guidelines, possibly reflecting differences in dietary patterns and public health priorities. For instance, Indonesia recommends a higher calcium intake for women aged ≥50 (1,200 mg) (19). In contrast, the Philippines, India, Japan, and Korea have lower recommended values (RDA or RNI) ranging from 600 to 800 mg per day (20–23). Similarly, vitamin D intake recommendations vary, with Taiwan, the Philippines, Korea, and Indonesia suggesting higher intakes for older women, while Japan maintains the same recommendation across all age groups (19, 20, 22–24). India's current guidelines, provided by the Indian Council of Medical Research, do not specify an RDA for vitamin D, instead advocating for sun exposure (outdoor activity) as the primary means of obtaining this nutrient (21). The only available dietary recommendation is supplementation with 10 µg of vitamin D for individuals with minimal sun exposure (21).

The consistency in recommendations for vitamins B1, B2, and B6 across most settings suggests general agreement on their importance, albeit with slight variations for older age groups in Korea and Japan (70 + or 75+, respectively) (22, 23). Vitamin B9 (folate) (RDA or RNI: 400 µg) and vitamin B12 (RDA or RNI: 2.4 µg) recommendations were consistent across Korea, Taiwan, and the Philippines, with lower values for Japan, Indonesia, and India (19–24). Magnesium intake recommendations also vary, with Taiwan and Indonesia recommending higher intakes than other settings (19, 24).

### 3.3 Reported dietary intake

Table 2 presents reported dietary intake levels of B-group vitamins, calcium, vitamin D, and magnesium for the national-level sources identified. There were significant variations in reported micronutrient intake among midlife women. In Taiwan, the average intake for vitamins B1, B2, and B3 met or exceeded RDAs across all age groups, though intake levels tended to be slightly lower among older adults (75+) (25). In contrast, the Philippines reported low proportions of adults meeting the Estimated Average Requirements (EAR) for vitamins B1 and B2, particularly in the oldest age groups (81 + years), whereas for vitamin B3 a higher proportion met EARs (26). Japan reported consistent intake levels across age groups, indicating stable consumption patterns (27). Data on B vitamin intake were not accessible for Indonesia or Korea.

**Table 2:** Reported dietary intake levels of B-group vitamins, calcium, vitamin D and magnesium, by region.

Region	Age group	Vitamin B1	Vitamin B2	Vitamin B3	Vitamin B6	Vitamin B9	Vitamin B12	Calcium	Vitamin D	Magnesium
Taiwan^a^ (25)	19–44	139%	126%	123%	117%	NA	227%	48%	49%	76%
	45–64	138%	126%	123%	125%	NA	178%	56%	45%	94%
	65–74	135%	117%	104%	112%	NA	150%	56%	40%	100%
	75+	121%	107%	86%	96%	NA	168%	52%	38%	82%
Philippines^b^ (26)	19–29	36.6	20.2	86.2	–	–	–	7.0	–	–
	30–39	30.2	14.9	83.4	–	–	–	7.3	–	–
	40–49	24.3	12.9	82.0	–	–	–	8.5	–	–
	50–59	20.4	11.1	78.7	–	–	–	6.2	–	–
	60–70	18.0	10.8	71.2	–	–	–	7.1	–	–
	71–80	12.5	8.9	63.9	–	–	–	6.5	–	–
	81+	10.4	7.8	46.0	–	–	–	7.0	–	–
	Adult women (19–59)	25.2	15.1	77.5	–	–	–	4.8	–	–
Indonesia		–	–	–	–	–	–	–	–	–
Japan^c^ (27)	20–29	0.77 mg	0.97 mg	25.6 NE mg	0.91 mg	226 µg	4.3 µg	408 mg	4.6 µg	192 mg
	30–39	0.83 mg	1.00 mg	26.6 NE mg	0.96 mg	233 µg	5 µg	406 mg	4.9 µg	205 mg
	40–49	0.89 mg	1.05 mg	28.6 NE mg	1.01 mg	247 µg	4.5 µg	441 mg	5.3 µg	219 mg
	50–59	0.83 mg	1.09 mg	27.9 NE mg	1.05 mg	284 µg	5.4 µg	472 mg	5.4 µg	233 mg
	60–69	0.93 mg	1.21 mg	30.3 NE mg	1.23 mg	328 µg	6.5 µg	539 mg	7.1 µg	269 mg
	70–79	0.94 mg	1.27 mg	30.5 NE mg	1.30 mg	348 µg	7.5 µg	574 mg	9.0 µg	275 mg
	80+	0.80 mg	1.11 mg	26.0 NE mg	1.09 mg	311 µg	6.2 µg	490 mg	7.4 µg	236 mg
S. Korea^c^ (28)	19–29	–	–	–	–	–	–	424.82 mg	2.46 µg	226.62 mg
	30–49	–	–	–	–	–	–	441.79 mg	2.87 µg	262.07 mg
	50–64	–	–	–	–	–	–	485.71 mg	2.73 µg	294.00 mg
	65–74	–	–	–	–	–	–	452.16 mg	2.49 µg	277.88 mg
	75+	–	–	–	–	–	–	370.94 mg	1.67 µg	241.79 mg
(29)	Premature menopause	1.19 mg	1.45 mg	11.44 mg	–	352.82 µg DFE^d^	–	540.84 mg	–	–
	Natural menopause	1.05 mg	1.55 mg	11.48 mg	–	354.89 µg DFE^d^	–	517.87 mg	–	–
(30)	Pre–menopause	–	–	–	–	–	–	463.80 mg	–	–
	Post–menopause	–	–	–	–	–	–	418.37 mg	–	–
India		–	–	–	–	–	–	–	–	–

a Values for Taiwan represent the average daily intake as a percentage of RDA.

b Values for the Philippines represent the proportion of the sample that met the estimated average requirement (EAR); values are averages for males and females (not split by gender), unless otherwise specified.

c Values for Korea and Japan are reported as average daily intakes.

d DFE, Dietary Folate Equivalents.

Reported calcium intake differed significantly. Japan and Korea reported low intakes (400–500 + mg) (27–30). Although Taiwan and the Philippines did not report specific intake levels, the proportions meeting intake recommendations were generally low across all age groups, with only −50% meeting RDAs in Taiwan and <10% meeting EARs in the Philippines (25, 26).

For vitamin D, Taiwan and Korea reported average daily intakes well below the recommended levels, with Taiwan <50% of the RDA for all age groups, especially for women >75 (38% of RDA) (25, 28). Japan's reported intake was closer to the recommended AI values, although these values were set lower than in other locations, particularly for older age groups (27).

Magnesium intake data were available for Taiwan, Korea, and Japan. In Taiwan, younger women (19–44) had lower average intake (76% of RDA) than older women (45–64; 65–74 years), where intake levels were closer to 100% of RDA (25). In Japan, average daily intake increased from younger to older age groups but remained slightly below RDAs overall; the largest gap was in younger women (20–29 years) (27). In Korea, average intake was generally below the RNI across age groups, particularly among older women (65–74; 75 + years) (28).

### 3.4 Serum levels and prevalence of deficiency

Supplementary Table S4 presents biomarker data (serum levels) and prevalence of deficiency/insufficiency for key micronutrients, where available. Within the available whole population-level datasets, data on micronutrient status or biomarker levels were scarce. Reported vitamin D status by age group was available only for Taiwan and the Philippines (25, 31). Taiwan also reported B-group vitamin status in the population based on mean serum levels and prevalence of deficiency and insufficiency (25). The incompleteness of publicly accessible data and heterogeneity in reporting presents challenges for identifying patterns of nutritional gaps or inadequacy, as well as limitations in cross-regional comparisons. Despite these limitations, the available biomarker data indicate a measurable burden of deficiency or borderline deficiency for selected micronutrients, particularly vitamin D and certain B-group vitamins, within age-defined midlife populations.

### 3.5 Associations with health outcomes

Next, we sought to identify links between micronutrient status and health outcomes. Table 3 summarizes potential relationships between the micronutrients of interest and outcomes in metabolic, musculoskeletal, cognitive, and mental health, as described in the publications reviewed. All associations reported reflect findings as described in the source literature and should be interpreted as observational associations.

**Table 3:** Relationship of micronutrients with selected health conditions.

Health domain	Health conditions or outcomes	Micronutrients^a^			
		B-group vitamins	Calcium	Vitamin D	Calcium & vitamin D
**Chronic inflammation and mobility**	Bone health	**Bone turnover markers** (Lower Vit B6 = Higher bone turnover [P1NP, CTX, Osteocalcin]) (32);	**BMD** (Lower calcium = Lower bone mineral density) (30, 34);	**BMD** (Lower Vit D = Lower bone mineral density) (37, 38);	**BMD** (Lower calcium and Vit D = Lower bone mineral density) (42–46);
		**Bone strength** (Higher Vit B9 and Vit B12 = increased bone mineral density and proximal femur strength) (33)	**Risk of osteoporosis** (Lower calcium intake = Higher osteoporosis risk.) (34);	**Femoral neck BMD** (Lower Vit D = Lower femoral neck BMD) (39);	**Bone mass loss** (Lower calcium and Vit D = Higher bone mass loss) (43);
		**Parathyroid hormone** (Lower Vit B6 = Higher serum PTH concentration) (32)	**Incidence of osteoporosis** (Lower calcium intake = Higher incidence of osteoporosis) (35);	**Total hip BMD** (Lower Vit D = Lower total hip BMD) (40);	**Bone turnover markers** (Lower calcium and Vit D = Higher bone turnover) (47, 48);
			**Bone health** (Lower calcium = Increased bone health risk) (36)	**Bone turnover markers** (Lower Vit D = Higher bone turnover markers [CTX-1, P1NP, osteocalcin]) (39);	**Incidence of hip fractures** (Lower calcium and Vit D = Higher incidence of hip fracture) (44);
				**Parathyroid hormone** (Lower Vit D = Higher PTH) (39);	**Bone loss** (Lower calcium and Vit D = Increased bone loss) (47)
				**Fragility hip fractures** (Lower Vit D = Higher risk of fragility hip fractures) (41);	
				**Vertebral fractures** (Lower Vit D = Higher risk of vertebral fractures) (41)	
	Muscle health		**Muscle loss** (Lower calcium = Muscle loss) (49);	**Hand grip strength** (Lower Vit D = Weak HGS) (51);	**Maintenance of muscle strength** (Lower calcium and Vit D = Reduced muscle strength) (48)
			**Sarcopenia** (Lower calcium = Higher sarcopenia) (50)	**Paraspinal muscle atrophy** (Lower Vit D = Increased paraspinal muscle atrophy) (51);	
				**Cross-sectional area of paraspinal muscle** (Lower Vit D = Reduced cross-sectional area of paraspinal muscle) (51);	
				**10-year fracture probability** (Lower Vit D = Higher 10-year fracture probability) (52)	
	Others		**Fall risk** (Lower calcium intake = Higher fall risk) (53);	**Physical performance** (Lower Vit D = Poor Short Physical Performance Battery score) (51);	
			**Tooth loss** (Lower calcium = Fewer teeth) (54)	**Lower back pain** (Lower Vit D = More intense lower back pain [Visual Analogue Score > 3]) (51)	
**Metabolic syndrome**	Blood pressure	**Hypertension** (Lower Vit B2 = Higher HTN) (55)			
	Lipids			**Fatty infiltration** (Lower Vit D = Higher fatty infiltration [ > Grade 2]) (51)	
	Diabetes	**Diabetes mellitus** (Lower Vit B2 = Higher diabetes risk) (55)			
	Cardiovascular	**Homocysteine** (Lower Vit B9 and or B12 = Higher homocysteine levels) (56, 57)	**Risk of CVD, stroke, myocardial infarction** (Lower calcium intake = Higher risk [for >10 postmenopausal years]) (58)	**Carotid intima-media thickness** (Lower Vit D = Increased C-IMT) (59)	
	Others	**Central obesity** (Lower Vit B2 = Presence of central obesity) (55);	**MetS** (Lower calcium = Risk of developing MetS) (29, 30)		
		**Prevalence of metabolic syndrome** (Lower Vit B2 = Higher MetS prevalence) (55)			
**Cognitive function and mental wellbeing**	Sleep				**Sleep quality**(Lower calcium and Vit D = Worse sleep quality [Pittsburgh Sleep Quality Index]) (60)
	Quality of life	**Health related quality of life** (Higher Vit B1/B2 = Better HRQOL) (61);	**Health related quality of life** (Lower calcium = Worse HRQOL) (61)		**Maintenance of quality of life** (Lower calcium and Vit D = Reduced Quality of Life) (48)
		**Problems with self-care, usual activities, pain, and discomfort** (Higher Vit B2 = Fewer problems) (61)			
**Other**	Burden of illness			**Burden of illness** Lower Vit D = Higher burden of illness (Cumulative Illness Rating Scale) (51, 62)	
	Reproductive and menopausal health	**Hot flushes severity** (Lower Vit B6 = Severe hot flushes) (63)	**Serum estrogen** (Lower calcium intake = Lower serum estrogen) (34)	**Vaginal tissue health** (Lower Vit D = Poor vaginal tissue health, measured by Vaginal Maturation Index) (62);	
				**Vaginal pH** (Lower Vit D = Higher vaginal pH [improved, more alkaline]) (62);	
				**Pain rating** (Lower Vit D = increased pain rating for vulvovaginal atrophy, as measure by Visual Analogue Scale) (62)	

This table presents reported relationships between micronutrient intake or status and health outcomes relevant to midlife women in the reviewed publications. For example, “Higher Vit B1/B2 = Better HRQoL” indicates that adequate or higher intake/status of the micronutrient(s) were associated with improved outcomes.

BMD, Bone Mineral Density; HRQOL, Health-Related Quality of Life; PTH, Parathyroid Hormone; CTX-1, C-terminal Telopeptide of Type I Collagen; P1NP, Procollagen Type I N-Terminal Propeptide; C-IMT, Carotid Intima-Media Thickness.

a Magnesium was a micronutrient of interest in this review but was omitted from this table as no publications were identified that reported relationships between magnesium intake/status and outcomes in the health domains of interest.

For B-group vitamins, several studies reported associations with metabolic health-related outcomes, including high blood pressure, type 2 diabetes mellitus, obesity, and metabolic syndrome (Table 3) (32). Low intake or low biomarker levels of these micronutrients were generally associated with adverse metabolic outcomes and reduced well-being. Higher intake of vitamins B1 and B2 was associated with higher health-related quality of life (HRQoL) scores and fewer problems related to self-care, usual activities, and pain/discomfort among premenopausal and postmenopausal women (33). Higher vitamin B6 intake was associated with reduced severity of hot flushes in midlife women (34). A study examining folate and vitamin B-12 status in postmenopausal Chinese-Singaporean women also found that higher circulating folate levels were positively associated with bone mineral density (BMD) and proximal femur strength, suggesting additional health benefits of these vitamins beyond metabolic outcomes (35).

For calcium and vitamin D, most studies focused on musculoskeletal health, particularly bone mineral density (BMD), risk or incidence of osteopenia and osteoporosis, bone fracture incidence or risk, and bone health markers (30, 36–51). Many studies also examined vitamin D and calcium in combination. Higher or adequate intake of calcium and vitamin D and higher serum 25-OHD levels were consistently associated with more favorable outcomes related to bone health. Beyond bone health, relationships between calcium and vitamin D and other health outcomes were also reported, including physical performance, grip strength, muscle health, and pain intensity; metabolic health markers; menopause-related symptoms; and HRQoL measures (Table 3) (29, 30, 33, 52–56).

## 4 Discussion

This review revealed substantial variability and gaps in the availability and type of micronutrient data for midlife women across Asia. The findings underscore the need for harmonized data collection and reporting practices to improve comparability and inform effective public health interventions within the region.

### 4.1 Current landscape of micronutrient status and intake in Asia: what do we really know?

Our review of the data landscape for micronutrients of interest revealed notable variation in several domains. Intake recommendations varied significantly in how DRIs are defined and presented. Some settings, such as Taiwan, adopt well-defined age categories (e.g., 19–29, 31–50 years), whereas others use broader classifications (e.g., DRIs for “adult women, 18+” in India) (Table 1). Additionally, variations in DRI parameters—e.g., RDAs, RNIs, or AIs—further complicate cross-region comparisons. While acknowledging that national recommendations are tailored to population-specific cultural, dietary, and environmental factors, such variation makes it challenging to identify trends and regional gaps.

For women in midlife, specific intake recommendations were often unclear or unavailable across the settings examined. Although women's micronutrient requirements change across midlife, especially from reproductive to post-reproductive stages, this was not consistently reflected in the available national-level guidance, apart from calcium. The lack of specific recommendations could hinder clear public health messaging on micronutrient requirements and other nutritional needs of midlife women.

Micronutrient intake data availability varied widely across the locations examined. Notably, calcium was the only micronutrient with population-level intake data reported across all locations, while data for other micronutrients varied (Table 2). Vitamin D status was more frequently reported than B-group vitamins or magnesium, despite their importance for women's health in midlife. As noted for DRIs, assessing intake adequacy proved challenging due to inconsistencies in age categories and/or the specific DRI parameters used (RDA/RNI vs. AI). In some cases, uncertainty in interpreting micronutrient adequacy levels arose even within a region due to discrepancies between age categories used for recommended and actual intake data.

Collecting and analyzing nutritional intake/status data, from dietary surveys to laboratory-based biomarker assessments, requires significant resources, especially in large, populous settings. Resource constraints determine national health priorities, leading to disparities in nutrition data availability across Asia, especially outside of the maternal/child health domain. Nevertheless, with population aging gaining importance (57), there is a growing need for preventive health intervention for midlife women across several domains, including metabolic, musculoskeletal, cognitive, and mental health (5, 7, 58, 59). Targeted, high-quality data collection and standardized data reporting are therefore needed to better guide national health policies and concerted transnational action for health.

### 4.2 Health impact and research gaps

Our findings highlight significant associations between micronutrient intake and several health outcomes in midlife women (Table 3), underscoring the importance of micronutrient adequacy. However, data gaps persist in Asia, where research has largely focused on calcium and vitamin D, with studies reporting associations between adequate intake and reduced fracture risk and improved musculoskeletal health. Conversely, research on other key micronutrients appears limited, with no studies on magnesium and six studies on B-group vitamins identified. Despite magnesium's importance in health and aging (60–62), none of the identified studies assessed the impact of its intake or adequacy within the health domains examined.

Beyond extensive research on calcium/vitamin D, few studies explored the impact of interactions among multiple micronutrients. One Japanese study reported association of deficiencies in multiple vitamins/minerals with incidence of bone fractures in post-menopausal women (63). We found no studies addressing the role of multiple micronutrient adequacy in Asian women in other health domains, although non-Asian studies exist. For example, a US National Health and Nutrition Examination Survey analysis (64) on fat-soluble vitamins (A, E, and D) revealed that vitamin D, specifically, was inversely associated with metabolic syndrome odds, with higher levels linked to improved metabolic health markers, adding another layer to understanding micronutrient impact on overall health. It was also reported that calcium, vitamin D, vitamin K, selenium, magnesium, and beta-carotene adequacy could be linked to higher BMD in postmenopausal women (65). Notably, existing literature primarily addresses micronutrient deficiencies rather than adequacy. While global studies on micronutrient deficiencies have been available for the past decade, comparable estimates for inadequate micronutrient intake were only recently published in 2024 (66, 67). Some settings, including Taiwan and Korea, reported both deficiency and borderline sufficiency rates for certain micronutrients, which informed national health messaging and recommendations (23, 25, 68). If resources permit, attention to addressing insufficiency or borderline deficiency of micronutrients in populations is warranted to inform policy and health promotion.

Overall, given the likelihood of inadequate intake of multiple micronutrients (67, 69), holistic strategies are needed to address micronutrient adequacy in midlife women. The complex interactions between micronutrients in skeletal health (e.g., calcium, vitamin D, vitamin K and magnesium) are now recognized (60, 70). Similar approaches should extend to other health domains, emphasizing optimal micronutrient proportions rather than individual nutrients. While deficiency prevention is necessary (e.g., avoiding scurvy from vitamin C deficiency), this alone does not ensure overall health or HRQoL. Emerging data indicates that optimal nutrient ratios, not merely adequate amounts, are important for health (70–72).

### 4.3 The case for more and better data

Given the scoping nature of this review, these findings reflect a descriptive mapping of heterogeneous evidence rather than harmonised estimates, and should be interpreted in light of variability in study design, populations, biomarkers, and reporting practices across data sources.

Overall, our findings highlight the need for more comprehensive, comparable, and accessible data on micronutrient intake and status across different populations in Asia. There is a clear need to harmonize nomenclature for reporting micronutrient intakes, deficiencies, and recommendations. This standardization would facilitate comparisons of nutritional status across studies and populations. Reliable, practical methods for quantifying micronutrient malnutrition are also needed for accurate diagnosis and effective interventions, particularly in older adult populations.

The availability of comprehensive data and detailed reports on micronutrient intake and/or status would facilitate needs assessment and development of targeted policies or interventions, as illustrated by public and multi-sector efforts in Korea and Taiwan.

For example, comprehensive data collection and reporting on approximately 20 micronutrients within Taiwan's Nutrition and Health Survey program facilitates comparisons to discern temporal trends and anticipate areas of changing or persisting needs (25, 73). For several micronutrients, intake as well as biomarker data (e.g., serum levels) are reported, providing a more complete picture of nutritional health within the population. Clear presentation of average intake relative to recommended intake by age and sex helps identify needs for specific groups within the population. For example, besides supporting calcium-focused health messaging for midlife and older women, the report highlighted the notable prevalence of deficiency in vitamin D, rates of marginal deficiency in vitamins B6 and B9, and lower-than-recommended magnesium intake among pre- and perimenopausal women (25). Importantly, easy access to such detailed public reports supports their use by policy-makers and multisectoral stakeholders to devise data-driven strategies that can address the specific nutritional needs of population groups, including midlife women.

Similarly, national-level data on health indicators, nutrient intake and nutritional biomarkers are collected regularly in the Korea National Health and Nutrition Examination Surveys (74). These data are actively analyzed to guide public health messaging and inform updates to national-level health policy and nutritional guidelines, including the Dietary Reference Intakes for Koreans (KDRIs) (68). Through Korea's demographic transition to an aged society, the KDRIs’ focus has shifted from addressing nutrient deficiencies to promoting overall health, preventing chronic diseases, and managing risks associated with both under-consumption and over-consumption of nutrients. The number of nutrients covered in the KDRIs has increased from 10 key nutrients in the 1960s to 40 nutrients (13 vitamins, 15 minerals) recognized as important for health (23). Through partnerships between the public sector and professional organizations such as the Korean Nutrition Society, recommendations are updated periodically based on recent scientific research and changes in the health status and dietary habits of the Korean population (68). Nationwide health screening and dietary guidelines for “new middle-aged” people focusing on adequate nutrition for healthier aging illustrate the use of data to inform targeted interventions and emphasize the importance of preparedness and self-care (75–77).

These examples highlight the value of collecting and utilizing comprehensive nutrition-related data for populations, and making it accessible to a range of stakeholders. On the other hand, Asia encompasses diverse populations with their unique cultural, economic, and health system burdens and priorities to consider. A recent publication on micronutrient deficiencies in India did not report data specific to midlife women, focusing instead on micronutrients deemed more critical for the general population and maternal, child and adolescent health, including folate, iodine, iron, and vitamin A (78). The study did report some deficiency data for vitamin D (approximately 60% overall) and vitamin B12 (50% deficiency, but with wide confidence intervals), illustrating the challenge of identifying, let alone addressing, the health needs of midlife women within current public health frameworks. Beyond established public health interventions such as government-mandated food fortification or supplementation programs delivered via local health centers, a broad array of strategies will be required (79, 80). These include public education multi-media campaigns to address fundamentals of nutrition or lifestyle modification, targeted strategies for midlife women as a group with distinct needs, and educational engagement sessions for healthcare professionals. In the public health space, targeted interventions may be limited to specific “issues” (e.g., breast cancer screening), whereas we believe that better integration and multi-sectoral collaborative approaches are needed. Collectively, the efforts of healthcare workers, policymakers, and communities at all levels will be needed to overcome nutritional barriers to health for midlife women.

During earlier stages of the demographic transition, investing in maternal/child health through data generation and action yielded gains in population health and lifespan. With these transitions continuing worldwide, preparedness for healthier ageing becomes ever more critical, especially for women, who are vulnerable to the impact of both menopause and aging on their health status and HRQoL (81). Midlife provides a window of opportunity for women to favorably influence their health trajectories by optimizing nutrition and other pillars of health (82).

### 4.4 Limitations

This review was limited by the scope and availability of data across the six Asian regions examined. National-level reports and peer-reviewed publications varied widely in methodology, definitions, and reporting formats, which limited direct comparability between regions. The literature search was restricted to English-language publications and publicly accessible national reports, which may have excluded relevant data available in local languages or unpublished offline sources. Additionally, the literature search was conducted up to August 2024; therefore, more recent studies were not included. As this was a scoping review, no formal risk of bias assessment was undertaken, and findings were synthesized descriptively. Accordingly the results provide a broad overview of the current evidence and should be interpreted as indicative rather than definitive. Nevertheless, this approach is appropriate for mapping emerging and fragmented evidence, integrating diverse data sources, and identifying key gaps to inform future research and public health priorities.

## 5 Conclusions

This scoping review addressed the question of what is known about micronutrient intake and status among midlife women in Asia, and how these relate to health outcomes during this life stage. We found variation in intake recommendations, reported intakes, and the prevalence of deficiency or insufficiency, alongside evidence suggesting associations between adequate micronutrient status and more favorable health outcomes and quality of life. Together, these findings underscore the importance of micronutrient adequacy in supporting health and well-being for women in Asia throughout midlife and the menopausal transition.

The primary contribution of this study lies in the systematic mapping of available population-level and peer-reviewed evidence, which highlights important gaps in data availability, consistency, and reporting across the region. By synthesising heterogeneous evidence using a scoping review approach, this work helps delineate current knowledge limitations and may inform future research priorities, nutritional surveillance efforts, and policy considerations related to healthy aging.

Despite their vulnerability to age-related health risks, this demographic group remains underrepresented in nutritional research and public health planning. Given finite resources and numerous potential interventions (public education, nutritional screening, dietary interventions, targeted supplementation), multi-sectoral collaboration is needed to ensure relevant and fit-for-purpose solutions.

Our findings highlight variation in micronutrient intake recommendations, actual intakes, prevalence of deficiency/insufficiency, and data availability across Asia. Recent research in Asian populations reinforces the associations between adequate micronutrient status/intake and more favorable outcomes for midlife women across health domains, as well as HRQoL. Beyond studying individual micronutrients, greater attention is needed on their interactions (including but not limited to vitamins D and K, calcium, and magnesium) across multiple domains, from cardiometabolic to musculoskeletal and cognitive health. This understanding could help address the challenge of “hidden hunger” in aging populations. For both individual women and populations, knowledge, preparedness, and timely intervention will be key to improving health and well-being.

## Data Availability

The original contributions presented in the study are included in the article/Supplementary Material, further inquiries can be directed to the corresponding author/s.
