# Supplementary material for: Micronutrient status among women in the midlife in selected Asian regions: a scoping review
Source: Front Glob Womens Health. 2026 May 21;7:1729971. doi: 10.3389/fgwh.2026.1729971 (PMC13233413; doi:10.3389/fgwh.2026.1729971)
Supplement: Supplementary file 1 [file Table1.docx]

Supplementary Material

## Supplementary Table 1. Design of scoping literature review – peer-reviewed publications

| **Included** | **Excluded** |
| --- | --- |
| **Populations/Participants** |  |
| Postmenopausal women (>12 months since last regular period)  Perimenopausal women  Women in midlife (approximate age range 40-60 years) | Child or adolescent studies  Studies on men or mixed age-group study populations where data for the target populations were not reported separately |
| General population of healthy individuals | Institutionalized individuals.  Individualized with serious late-stage or terminal conditions (e.g., cancer). |
| **Concepts** |  |
| Micronutrient intake  Studies focusing on micronutrients of interest (B-group vitamins, calcium, vitamin D, and magnesium) | Studies focusing on micronutrients other than those of interest |
| Micronutrient status  Studies focusing on prevalence of micronutrient status, adequacy or deficiency for micronutrients of interest (B-group vitamins, calcium, vitamin D, magnesium) and their associated biomarkers, e.g., serum 25-hydroxyvitamin D. | Studies focusing on micronutrients other than those of interest |
| Health impact of micronutrient intake or micronutrient status  Studies on relationships between micronutrient intake or status and risk, incidence/prevalence, severity of selected health outcomes and conditions that are important during midlife, e.g., metabolic syndrome, cardiovascular disease risk factors, osteoporosis, menopausal symptoms. | Studies reporting health outcomes without reference to individuals’ micronutrient intake or micronutrient status |
| **Context** |  |
| Asian populations (regions of interest: India, Indonesia, Japan, the Philippines, South Korea, Taiwan) | Studies in Asian regions other than the regions of interest |
| **Types of studies** |  |
| Studies reporting primary research findings (selected types):   - Observational studies, including cross-sectional, cohort/longitudinal studies, and surveys - Interventional studies, e.g., randomized controlled trials - Systematic reviews of primary research studies | - Pilot studies - Case-control studies - Case reports or case series - Genetic or genomic studies |
| **Other criteria** |  |
| - Studies in humans - Publications in English - Abstracts available - Published from 01-Jan-2013 to 13-Aug- 2024 (date of search) | - Animal studies - Non-English publications - Preprints or retracted publications - Narrative review articles, letters to the editor, editorials, commentaries - Published before 01-Jan-2013 or after 13-Aug-2024 (date of search) |

**Supplementary Table 2. Example search strategy for peer-reviewed publications**

| **Structure of search string:**  [Micronutrient-related terms] AND [Population-related terms] AND [Context-related terms] AND [Health outcomes related terms] |
| --- |
| **Example search string:**  (Magnesium[Title/Abstract] OR Magnesium[Mesh] OR "Magnesium Deficiency"[Mesh])  AND  ("Nutritional Status"[Mesh] OR "Diet"[Mesh] OR "Dietary Supplements"[Mesh] OR (dietary intake) OR status OR deficiency OR insufficiency OR (serum concentration) OR (plasma concentration) OR (serum level) OR (blood concentration))  AND  ("Perimenopause"[MeSH Terms] OR "Menopause"[MeSH Terms] OR "Postmenopause"[MeSH Terms] OR "perimenopaus*"[All Fields] OR "menopaus*"[All Fields] OR "postmenopaus*"[All Fields]) AND "female"[MeSH Terms]  AND  ("Asia"[Mesh] OR China OR Japan OR Korea OR India OR Indonesia OR Malaysia OR Philippines OR Singapore OR Taiwan OR Thailand OR Vietnam OR "Southeast Asia" OR "East Asia" OR "South Asia" OR "Asia Pacific")  AND  ("metabolic syndrome" OR "cardiovascular disease" OR obesity OR dyslipidemia OR diabetes OR osteoporosis OR osteoarthritis OR (cognitive function) OR cognition OR anxiety OR depression OR "sleep disorder" OR "quality of life" OR (menopausal symptoms)) |

## Supplementary Table 3. Scheme for data extraction

| **Data extraction scheme**  Micronutrients of interest: B-group vitamins, calcium, vitamin D, and magnesium  Data extracted  - Aim(s) of the study  - Study design  - Location where the study was done  - Age range of study participants  - Menopausal status (if reported): post-menopausal, peri-menopausal or pre-menopausal  - Details of micronutrient intake levels, e.g., mg/day  - Prevalence of micronutrient deficiencies by age group  - Details of micronutrient supplementation, type and dose  - Any comparators or comparisons between subgroups  - Number of study participants  - Study or follow-up duration  - Treatment duration  - Main outcomes analyzed  - Key findings and conclusion  - Other details relevant to the micronutrients of interest |
| --- |

## Supplementary Table 4. Reported serum levels, deficiency or marginal deficiency of B-group vitamins, calcium, vitamin D and magnesium, by region.

| **Reported Serum Levels** | | | | | | | | | |
| --- | --- | --- | --- | --- | --- | --- | --- | --- | --- |
| **Region** | **Age group** | **Vitamin B1** | **Vitamin B2** | **Vitamin B6** | **Vitamin B9** | **Vitamin B12** | **Calcium** | **Vitamin D** | **Magnesium** |
| **Taiwan [25]** | 19-44 | 43.7 (1.20) | 23.6 (1.24) | 66.9 (4.41)  nM^1^ | 10.1 (0.21) ng/ml ^2^ | 392.3 (10.13) pmol/L ^3^ | 9.31 (0.02) mg/dL | 22.1 (0.42) ng/mL | 2.08 (0.01) mg/dL ^4^ |
|  | 45-64 | 51.4 (1.70) | 30.5 (1.47) | 99.9 (4.95)  nM^1^ | 13.4 (0.35) ng/ml ^2^ | 507.8 (13.56) pmol/L ^3^ | 9.37 (0.02) mg/dL | 27.6 (0.49) ng/mL | 2.15 (0.01) mg/dL ^4^ |
|  | 65-74 | 55.2 (2.07) | 35.7 (2.39) | 113.7 (7.84)  nM^1^ | 14.2 (0.32) ng/ml ^2^ | 584.7 (18.87) pmol/L ^3^ | 9.33 (0.02) mg/dL | 30.5 (0.55) ng/mL | 2.15 (0.01) mg/dL ^4^ |
|  | 75+ | 59.5 (3.58) | 39.8 (4.51) | 101.7 (7.38)  nM^1^ | 14.1 (0.60) ng/ml ^2^ | 607.0 (40.61) pmol/L ^3^ | 9.30 (0.03) mg/dL | 33.0 (1.00) ng/mL | 2.15 (0.02) mg/dL ^4^ |
| **Philippines [31]** | 20-39 | - | - | - | - | - | - | 63.5 ± 1.7 nmol/ml | - |
|  | 40-59 | - | - | - | - | - | - | 70.3 ± 1.1 nmol/ml | - |
|  | 60+ | - | - | - | - | - | - | 77.1 ± 1.9 nmol/ml | - |
| **Indonesia** |  | - | - | - | - | - | - | - | - |
| **Japan** |  | - | - | - | - | - | - | - | - |
| **S. Korea** |  | - | - | - | - | - | - | - | - |
| **India** |  | - | - | - | - | - | - | - | - |
| **Reported Percentage with Deficiency or Marginal/Borderline Deficiency** | | | | | | | | | |
| **Region** | **Age group** | **Vitamin B1** | **Vitamin B2** | **Vitamin B6** | **Vitamin B9** | **Vitamin B12** | **Calcium** | **Vitamin D** | **Magnesium** |
| **Taiwan [25]** | 19-44 | - | - | 12.2%; 16.0% | 0% †; 10.5% ‡ | 3.0% † | - | 42.9% †;  42.3% ‡ | - |
|  | 45-64 | - | - | 7.8%; 9.2% | 0% †; 4.2% ‡ | 2.0% † | - | 20.5% †;  40.3% ‡ | - |
|  | 65-74 | - | - | 9.6%; 9.0% | 0% †; 1.6% ‡ | 2.2% † | - | 15.1% †;  34.5% ‡ | - |
|  | 75+ | - | - | 11.7%; 11.1% | 0% †; 2.2% ‡ | 2.4% † | - | 11.3% †;  31.4% ‡ | - |
| **Philippines [31]** | 20-39 | - | - | - | - | - | - | 31.9% †;  41.5% ‡ ^1^ | - |
|  | 40-59 | - | - | - | - | - | - | 16.1% †;  46.7% ‡ ^1^ | - |
|  | 60+ | - | - | - | - | - | - | 9.3% †;  42.3% ‡ ^1^ | - |
| **Indonesia** |  | - | - | - | - | - | - | - | - |
| **Japan** |  | - | - | - | - | - | - | - | - |
| **S. Korea** |  | - | - | - | - | - | - | - | - |
| **India** |  | - | - | - | - | - | - | - | - |

^1^ Marginal B6 deficiency is defined as 20 nM ≤ B6 <30 nM and deficiency as <20 nM.

^2^ Marginal folate deficiency is defined as 3 ng/mL ≤ folate <6 ng/mL and deficiency as <3 ng/mL.

^3^ B12 deficiency is defined as <148 pmol/L

^4^ Reference range of normal blood magnesium value: 1.7-2.2 mg/dL for 13-20 years old, 1.6-2.6 mg/dL for 21-59 years old, 1.6 for 60-90 years old -2.4 mg/dL.

NR, not reported

† Deficiency

‡ Borderline deficiency or insufficiency

^1^ Manila Metro area
